# Two-locus identity coefficients in pedigrees

**DOI:** 10.1093/g3journal/jkac326

**Published:** 2022-12-16

**Authors:** Magnus Dehli Vigeland

**Affiliations:** Department of Medical Genetics, Oslo University Hospital and the University of Oslo, PO Box 4950 Nydalen, 0424 Oslo, Norway

**Keywords:** pairwise relatedness, identity-by-descent, identity coefficients, kinship, pedigree analysis, two-locus coefficients, expected likelihood ratio, realized relatedness, linkage

## Abstract

This paper proposes a solution to a long-standing problem concerning the joint distribution of allelic identity by descent between two individuals at two linked loci. Such distributions have important applications across various fields of genetics, and detailed formulas for selected relationships appear scattered throughout the literature. However, these results were obtained essentially by brute force, with no efficient method available for general pedigrees. The recursive algorithm described in this paper, and its implementation in R, allow efficient calculation of two-locus identity coefficients in any pedigree. As a result, many existing procedures and techniques may, for the first time, be applied to complex and inbred relationships. Two such applications are discussed, concerning the expected likelihood ratio in forensic kinship testing, and variances in realized relatedness.

## Introduction

The study of genetic relatedness centers around various coefficients of relatedness, each defined as the probability that certain alleles are *identical by descent* (IBD), i.e. that they originate from the same ancestral allele within the given pedigree. For the alleles of two individuals at a single locus, common coefficients range in complexity from the simple kinship and inbreeding coefficients (Wright 1922) to the detailed identity coefficients which characterize the distribution of IBD states for any pairwise relationship (Jacquard 1974).

The generalization of IBD probabilities to multiple linked loci was pioneered by Haldane (1949), who defined two-locus kinship and inbreeding coefficients and derived explicit formulas in special cases. Seeking a general procedure, he admitted defeat in the case of individuals with related common ancestors. (In fact, the problems Haldane faced here are unsolvable in his formulation, as we demonstrate in Section “Examples.”) Weir and Cockerham (1969) outlined a more general method, but their approach is impractical except in small pedigrees. Finally, Thompson (1988) proposed an efficient, recursive algorithm for two-locus kinship coefficients, elucidated and popularized by Weeks and Lange (1992). This algorithm is implemented in the MORGAN software (https://sites.stat.washington.edu/thompson/Genepi/MORGAN/Morgan.shtml) and also included in the R package ribd featured in the present paper. The latter has the advantage of supporting selfing and pedigrees with inbred founders (Vigeland 2020).

Just as in the single-locus case, the two-locus kinship coefficient is only the simplest in a range of two-locus coefficients. For noninbred pairs, the 9 two-locus IBD coefficients $\kappa_{ij}(\rho )$, for i,j∈{0,1,2}, are defined as the probabilities of sharing exactly *i* alleles IBD at the first locus and *j* at the second, where *ρ* denotes the recombination fraction. These coefficients, and their extension to inbred relationships, are the focus of the present article.

Two-locus IBD coefficients have played central roles in a variety of applications over the years. For example, they were important in the development of medical linkage analysis (Bishop and Williamson 1990) and quantitative-trait linkage analysis (Almasy and Blangero 1998). Applications in forensic genetics include match probabilities (Buckleton and Triggs 2006; Bright *et al.* 2013), kinship testing (Egeland and Sheehan 2008; Egeland and Slooten 2016) and mixture analysis (Dørum *et al.* 2015) among others. Furthermore, two-locus coefficients encode key information about distributions of *realized* (or *actual*, or *genomic*) *relatedness*, which is sometimes more relevant than the pedigree-based expectation (Speed and Balding 2015). A considerable body of literature is devoted to estimating variances in realized IBD sharing by integrating suitable two-locus IBD coefficients (Guo 1995, 1996; Hill and Weir 2011, 2012; Thompson 2013).

Despite the enduring interest in two-locus IBD coefficients, no efficient algorithm for their calculation has hitherto been described in the literature. Formulas for $\kappa_{ij}(\rho )$ in special cases were given by Denniston (1975) using a path-counting approach, and subsequent authors have used similar, direct methods to analyze certain classes relationships. Nevertheless, previous works involving two-locus IBD coefficients (or closely related probabilities) have been invariably limited to simple, noninbred relationships.

In this paper, we propose and implement a method for computing pairwise joint two-locus IBD probabilities in any pedigree. This includes the 9 coefficients $\kappa_{ij}(\rho )$ for noninbred pairs, and also the 81 two-locus condensed identity coefficients for general pairs. The key ingredient is a recursive algorithm for *generalized two-locus kinship coefficients*, inspired by previous works on single-locus identity coefficients (Weeks and Lange 1988; Lange and Sinsheimer 1992). Using this method, and its implementation in the R package ribd (Vigeland 2020), several existing applications may be extended to general pedigrees, including inbred relationships. Two such applications are explored in the Discussion; one in forensic kinship testing and one in the study of variances in realized relatedness.

## Background

In this section, we review the basics of relatedness coefficients and settle our notation.

A *pairwise relationship* is a triple (a,b,P), where P is a finite, connected pedigree, and *a* and *b* are (not necessarily distinct) members of P. We will usually assume that the relationship is *nontrivial*, i.e. that *a* and *b* are connected with at least one path in P, although most of the results also hold in the trivial case. Homologous alleles of *a* and *b* are IBD if they descend from the same allele carried by a common ancestor of *a* and *b* within P. We often suppress P in our notation, but emphasize that any calculations or concepts based on IBD only make sense in the context of an explicit pedigree. We restrict our attention to diploid species.

### Single-locus coefficients

The *kinship coefficient*$\varphi^{ab}$ between *a* and *b* is the probability that a random allele from *a* is IBD with a random allele from *b* at the same autosomal locus. The *inbreeding coefficient*$f^{c}$ of a child *c* between *a* and *b* is defined by $f^{c}=\varphi^{ab}$. We say that *c* is *inbred* if $f^{c}>0$, and *completely inbred* if $f^{c}=1$. Pedigree founders are usually assumed to be noninbred, but this may be relaxed in some applications (Vigeland 2020).

*Note about notation*: Relatedness coefficients are conventionally written with the individuals as subscripts rather than superscripts. We use superscripts in this paper, reserving subscripts for other indices. When the context is clear, we may drop the superscripts and simply write *φ* or *f*.

**Fig. 1.**
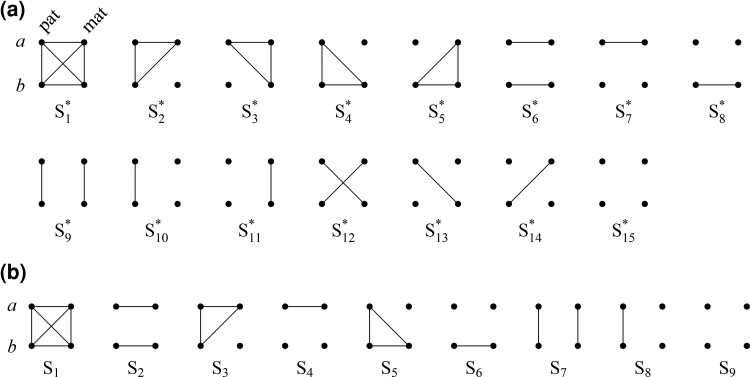
Single-locus identity states. Each diagram depicts the four homologous alleles carried by *a* and *b* at an autosomal locus, where IBD alleles are connected with a line segment. a) Detailed states, with paternal alleles to the left and maternal alleles to the right. b) Condensed states ignoring the paternal/maternal ordering.

Figure 1a shows all possible patterns of IBD between the four alleles carried by two individuals at a single autosomal locus. The 15 patterns $S_{1}^{*},\ldots ,S_{15}^{*}$ are called the (single-locus) *detailed identity states*. The expected relative frequencies of these states in a given relationship (a,b,P) are denoted $\delta_{1},\ldots ,\delta_{15}$, and referred to as the *detailed identity coefficients* of *a* and *b*.

If the allele ordering (i.e. the paternal/maternal origin) within each individual is ignored, the 15 states are reduced to 9 *condensed identity states*$S_{1},\ldots ,S_{9}$ (Fig. 1b), with expected relative frequencies $\Delta_{1},\ldots ,\Delta_{9}$. The notation and ordering follow Jacquard (1974).

### Noninbred relationships

We say that a relationship R=(a,b,P) is *noninbred* if neither *a* nor *b* is inbred in P. Note, however, that other members of P may be inbred. For noninbred relationships, only $\Delta_{9},\Delta_{8},\Delta_{7}$ may be nonzero—the probabilities that *a* and *b* share, respectively, 0, 1, and 2 alleles IBD. Following Thompson (1975), we denote these by $\kappa_{0},\kappa_{1},\kappa_{2}$. We refer to them as *IBD coefficients*, to distinguish them from the previously defined identity coefficients.

A noninbred relationship R is called *unilineal* if $\kappa_{2}=0$, and *bilineal* if $\kappa_{2}>0$ (Cotterman 1940). Furthermore, R is *direct* if *a* is a direct descendant of *b* or *vice versa*, and *collateral* otherwise. The following simple fact will be used in later proofs:

Lemma 1 *Let R=(a,b,P) be a noninbred relationship with a≠b. Then the following implications hold: Risbilineal⟹Riscollateral⟹a,bare nonfounders.*

Proof. For the first implication, suppose for a contradiction that R is bilineal and that *a* is an ancestor of *b*. If f,m are the parents of *b*, we can then assume w.l.o.g. that *a* is an ancestor of (or equal to) *f*. In order for $\kappa_{2}$ to be nonzero, *a* must be related to *m* as well. But then *m* and *f* are related, contradicting the assumption that *b* is noninbred. The same argument applies if *b* is an ancestor of *a*, hence we conclude that R cannot be direct.

The second implication follows from the fact that if *a* is a founder of P, the only relatives of *a* in P are the descendants of *a*. ȃ□

Finally, we recall the close connection between *φ* and *κ* at a single locus. The following well-known facts, which we include for easy reference, are straightforward from the definitions (see also Thompson 2000).

Proposition 1 *Let R=(a,b,P) be noninbred, with kinship coefficient *φ* and IBD coefficients $\kappa =(\kappa_{0},\kappa_{1},\kappa_{2})$*.

- *For any R, *φ* is determined by *κ*, by the formula $\varphi =\frac{1}{4}\kappa_{1}+\frac{1}{2}\kappa_{2}$.*
- *If R is unilineal, then *κ* is determined by *φ*, by the formula κ=(1−4φ,4φ,0).*
- *If R is bilineal, then *κ* is determined by *φ* together with the parental kinships. If f,g are the parents of *a* and m,n the parents of *b* (cf. Lemma 1), then:*
  (1) $$\begin{array}{rl} \kappa_{2} & =\varphi^{\;fm}\varphi^{gn}+\varphi^{\;fn}\varphi^{gm},\\ \kappa_{1} & =4\varphi -2\kappa_{2},\\ \kappa_{0} & =1-\kappa_{1}-\kappa_{2}. \end{array}$$

### Two-locus coefficients

All of the single-locus coefficients described above can be generalized to multiple loci by considering the *joint* IBD probabilities at the loci. Crucially, such multilocus coefficients are functions of the recombination rates between the loci. In the present paper, we restrict our attention to two autosomal loci, $L_{1}$ and $L_{2}$, with recombination rate ρ∈[0,0.5], which we assume to be the same for males and females.

The *two-locus kinship coefficient*$\varphi_{11}^{ab}(\rho )$ is the probability that a random gamete from *a* and a random gamete from *b* contain IBD alleles at both $L_{1}$ and $L_{2}$. If *a* and *b* are clear from the context, we will write this coefficient as $\varphi_{11}(\rho )$, or simply $\varphi_{11}$, with the understanding that it is always a function of *ρ*. As first observed by Haldane (1949), we have $\varphi_{11}(0)=\varphi$ and $\varphi_{11}(0.5)=\varphi^{2}$ for any relationship.

If both *a* and *b* are noninbred, we define the *two-locus IBD coefficient*$\kappa_{ij}^{ab}(\rho )$, for i,j∈{0,1,2}, to be the probability that *a* and *b* share exactly *i* IBD alleles at $L_{1}$ and exactly *j* IBD alleles at $L_{2}$. As with $\varphi_{11}$, we may drop *a*, *b* and *ρ* from the notation and simply write $\kappa_{ij}$. Clearly, $\kappa_{ij}=\kappa_{\;ji}$ so the coefficients form a symmetric matrix:

$$K(\rho )=\left(\begin{array}{ccc} \kappa_{00} & \kappa_{01} & \kappa_{02}\\ \kappa_{10} & \kappa_{11} & \kappa_{12}\\ \kappa_{20} & \kappa_{21} & \kappa_{22} \end{array}\right).$$

If ρ=0, the absence of recombination implies that any pedigree path between *a* and *b* yields IBD alleles either at both or none of the loci. Hence $\kappa_{ii}(0)=\kappa_{i}$ for i=0,1,2 and $\kappa_{ij}(0)=0$ if i≠j. At the other extreme, ρ=0.5, the two loci segregate independently of each other, thus $\kappa_{ij}(0.5)=\kappa_{i}\kappa_{j}$ for all i,j∈{0,1,2}.

Another important observation is that for any *ρ*, the row and column sums of K(ρ) are *κ*:

(2) $$\kappa_{i0}+\kappa_{i1}+\kappa_{i2}=\kappa_{0i}+\kappa_{1i}+\kappa_{2i}=\kappa_{i},\;\;for\;alli=0,1,2.$$

Indeed, this is a simple consequence of the law of total probability applied to the first locus (row sums) and the second locus (column sums).

The relations (2) are especially powerful for unilineal relationships, where $\kappa_{2}=0$. Then also $\kappa_{i2}=\kappa_{2i}=0$ for i=0,1,2, and we obtain after simplification,

(3) $$K=\left(\begin{array}{ccc} 1-2\kappa_{1}+\kappa_{11} & \kappa_{1}-\kappa_{11} & 0\\ \kappa_{1}-\kappa_{11} & \kappa_{11} & 0\\ 0 & 0 & 0 \end{array}\right).$$

Since $\kappa_{1}=\kappa_{11}(0)$, it follows that in the unilineal case, the entire matrix *K* is uniquely determined by $\kappa_{11}$.

Finally, we generalize to the case where *a* and *b* may be inbred. For i,j∈{1,…,9} we define the *two-locus identity coefficient*$\Delta_{ij}=\Delta_{ij}^{ab}(\rho )$ as the probability that the condensed identity states at $L_{1}$ and $L_{2}$ are $S_{i}$ and $S_{j}$, respectively. For any fixed *ρ* these coefficients form a symmetric 9×9 matrix:

(4) $$D=\left(\begin{array}{ccc} \Delta_{11} & \cdots & \Delta_{19}\\ \vdots & \ddots & \vdots \\ \Delta_{91} & \cdots & \Delta_{99} \end{array}\right).$$

As in the noninbred case, the row sums, and also the column sums, of *D* are the single-locus coefficients $\Delta_{1},\ldots ,\Delta_{9}$.

### Examples

The purpose of this section is to illustrate some of the complications that arise with linked loci.

In light of Proposition 1, it is natural to wonder if there exists a direct relationship between the two-locus coefficients $\varphi_{11}$ and *K*. Especially for unilineal relationships, where the one-locus situation boils down to the formula $\kappa_{1}=4\varphi$, one might hope for a similar identity relating $\kappa_{11}$ and $\varphi_{11}$. Unfortunately, as the following two examples demonstrate, no such formula can exist.

The first well-known example (e.g. Section 4.5 of Thompson 2000) shows that two relationships may have different $\kappa_{11}$, but the same $\varphi_{11}$. An interpretation of this is that the relationships are theoretically distinguishable given genetic data from the two individuals, but *not* with data from their child alone.

Example 1 **(Different $\kappa_{11}$, same $\varphi_{11}$).**

*The relationships of grandparent–grandchild (G) and half-siblings (H) have different two-locus IBD functions,*

(5) $$\begin{array}{rl} & \kappa_{11}^{G}=\frac{1}{2}\bar{\rho },\\ & \kappa_{11}^{H}=\frac{1}{2}(\rho^{2}+{\bar{\rho }}^{2}), \end{array}$$

*where $\bar{\rho }=1-\rho$, but identical two-locus kinship, $\varphi_{11}^{G}=\varphi_{11}^{H}=\frac{1}{8}\bar{\rho }(\rho^{2}+{\bar{\rho }}^{2})$. These functions are easily verifiable by direct calculation.*

Next, we show that the opposite situation is also possible. This particular example appears to be original, although it seems likely that the effect it illustrates has been known to previous authors.

Example 2 **(Same $\kappa_{11}$, different $\varphi_{11}$).**

*Consider an outbred parent-offspring relationship (PO), and compare it with half-sibs whose shared parent is completely inbred (H-i). It is easy to see that both of these satisfy* $\kappa_{11}(\rho )=1$ *for all* *ρ*; *in other words, they have a constant IBD matrix*

(6) $$K^{\left(\mathrm{PO}\right)}=K^{\left(H-i\right)}=\left(\begin{array}{ccc} 0 & 0 & 0\\ 0 & 1 & 0\\ 0 & 0 & 0 \end{array}\right).$$

In the H-i case, the IBD alleles are always *in cis*, i.e. on the same haplotype, since they come from the same parent. For PO, on the other hand, the alleles are *in cis* in the child, but not necessarily in the parent. This difference leads to distinct $\varphi_{11}$ functions (see Example 4 for calculations):

(7) $$\begin{array}{rl} \varphi_{11}^{\left(\mathrm{PO}\right)} & =\frac{1}{4}\bar{\rho }(\rho^{2}+{\bar{\rho }}^{2}),\\ \varphi_{11}^{\left(H-i\right)} & =\frac{1}{4}{\bar{\rho }}^{2}. \end{array}$$

A remarkable consequence of Example 2 is that PO and H-i cannot be distinguished by means of (unphased) genetic data from the two individuals themselves, but can be so given data from their child alone.

For our final example, we return to Haldane’s problem mentioned in the introduction. In our notation this amounts to the following: Given a relationship (a,b,P) where *a* and *b* have two different common ancestors P,Q in P, find a formula for $\varphi_{11}^{ab}$ expressed by the single-locus coefficient $\varphi^{PQ}$ and the two-locus coefficient $\varphi_{11}^{PQ}$ between the ancestors. Failing to do so, Haldane remarked:

*It is possible that [these coefficients] do not give all the needful information.*

As it turns out, Haldane’s intuition was correct: In general, $\varphi_{11}^{ab}$ depends not only on $\varphi^{PQ}$ and $\varphi_{11}^{PQ}$, but also on the two-locus IBD matrix $K^{PQ}$. Here is an illustration:

Example 3  

*Suppose *a* and *b* are full siblings whose parents *P* and *Q* are unilineally related. Then the two-locus kinship $\varphi_{11}$ of *a* and *b* is given by*

(8) $$\varphi_{11}=(\rho^{2}+{\bar{\rho }}^{2})\frac{{\bar{\rho }}^{2}}{4}+\frac{{\bar{\rho }}^{2}}{2}\varphi_{11}^{PQ}+\bar{\rho }\rho \varphi^{PQ}+\frac{\rho^{2}}{8}+\frac{\rho^{2}}{32}\kappa_{11}^{PQ}.$$

*In particular,* $\varphi_{11}$ *cannot be expressed solely by* $\varphi^{PQ}$ *and* $\varphi_{11}^{PQ}$ .

The formula (8) is obtained as follows. The first two terms cover the cases where the emitted gametes are nonrecombinant, either originating from the same parent (probability $(\rho^{2}+{\bar{\rho }}^{2})(\frac{1}{2}\bar{\rho }{)}^{2}$) or one from each ($2\varphi_{11}^{PQ}(\frac{1}{2}\bar{\rho }{)}^{2}$). The middle term $\bar{\rho }\rho \varphi^{PQ}$ is the probability of IBD at both loci when one gamete is recombinant and the other not. Finally, when both gametes are recombinant, the alleles may come from the same parent at each locus, i.e. paternal alleles at $L_{1}$ and maternal at $L_{2}$, or vice versa (total probability $\frac{1}{8}\rho^{2}$), or one from each parent at each locus ($\frac{\rho^{2}}{32}\kappa_{11}^{PQ}$). Altogether, this gives the claimed formula.

For an explicit example, consider the case of siblings whose parents are half-siblings. Inserting the expressions for $\varphi_{11}^{H}$ and $\kappa_{11}^{H}$ from Example 1 into (8), we obtain after simplification:

$$\varphi_{11}=\frac{1}{64}(8\rho^{6}-40\rho^{5}+118\rho^{4}-194\rho^{3}+177\rho^{2}-80\rho +20).$$

### Phased components of two-locus coefficients

Denniston (1975) introduced a refinement of the $\kappa_{ij}$ coefficients, taking into account the *phase* of the IBD alleles. He found efficient formulas for some of these extended coefficients, but had to resort to tedious path tracing for the remaining ones. One contribution of the current paper is to enable recursive calculation of *all* of these phased coefficients, which in turn provide the $\kappa_{ij}$’s.

Starting with the coefficient $\kappa_{11}$, this naturally splits into four phased components:

$$\kappa_{11}=\kappa_{11}^{cc}+\kappa_{11}^{tc}+\kappa_{11}^{ct}+\kappa_{11}^{tt}.$$

**Fig. 2.**
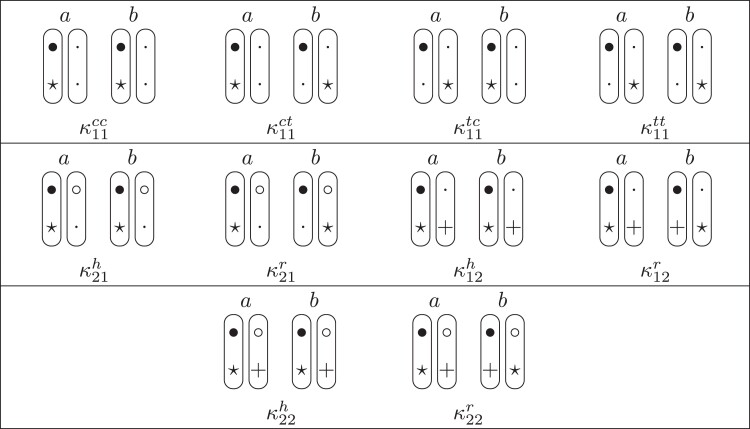
Phased IBD patterns underlying the coefficients $\kappa_{11}$, $\kappa_{21}$, $\kappa_{12},$ and $\kappa_{22}$. Each diagram shows phased (but unordered) haplotypes of *a* and *b* at two loci. Equal symbols represent IBD alleles, except for the tiny dots, which are not IBD with any other. Mnemonic superscripts: c (*cis*), t (*trans*), h (haplotype), and r (recombination). *Top row*: The four possible cis/trans combinations when *a* and *b* share exactly one IBD allele at each locus. *Middle row*: Configurations with two IBD alleles at one locus and one at the other. *Bottom row*: Configurations with two IBD alleles at each locus.

The superscripts signify if the IBD alleles are *in cis* or *in trans* in *a* and b, respectively. The underlying IBD patterns are shown in the top row of Fig. 2.

Turning to bilineal relationships, the coefficients $\kappa_{21}$, $\kappa_{12},$ and $\kappa_{22}$ have similar decompositions:

(9) $$\begin{array}{rl} \kappa_{21} & =\kappa_{21}^{h}+\kappa_{21}^{r},\\ \kappa_{12} & =\kappa_{12}^{h}+\kappa_{12}^{r},\\ \kappa_{22} & =\kappa_{22}^{h}+\kappa_{22}^{r}, \end{array}$$

where the superscripts indicate whether the IBD alleles form the same haplotype(s) in *a* and *b*, or if a recombination has happened. See Fig. 2 (rows 2 and 3) for illustrations. Our notation differs slightly from that of Denniston (1975).

For each pattern in Fig. 2, it is straightforward to find the probability that *a* and *b* emit gametes with IBD alleles at both loci. For instance, in the diagram corresponding to $\kappa_{11}^{cc}$ (top-left), this amounts to $(\frac{1}{2}\bar{\rho }{)}^{2}$, since both individuals must emit the same haplotype unrecombined. With similar calculations for the other patterns in the top row, we finally obtain a two-locus analogue of the single-locus formula $\varphi =\frac{1}{4}\kappa_{1}$ (Proposition 1b) for unilineal relationships:

(10) $$\varphi_{11}=\frac{{\bar{\rho }}^{2}}{4}\kappa_{11}^{cc}+\frac{\rho \bar{\rho }}{4}(\kappa_{11}^{ct}+\kappa_{11}^{tc})+\frac{\rho^{2}}{4}\kappa_{11}^{tt}.$$

For bilineal relationships, we must include all the patterns in Fig. 2, producing the formula

(11) $$\begin{array}{rl} \varphi_{11}= & \frac{R}{2}\kappa_{22}^{h}+\frac{\rho \bar{\rho }}{2}\kappa_{22}^{r}+\frac{R}{4}(\kappa_{21}^{h}+\kappa_{12}^{h})+\frac{\rho \bar{\rho }}{4}(\kappa_{21}^{r}+\kappa_{12}^{r})+\frac{{\bar{\rho }}^{2}}{4}\kappa_{11}^{cc}\\ & +\frac{\rho \bar{\rho }}{4}(\kappa_{11}^{ct}+\kappa_{11}^{tc})+\frac{\rho^{2}}{4}\kappa_{11}^{tt}, \end{array}\;$$

where $R=\rho^{2}+{\bar{\rho }}^{2}$. Exploiting the symmetries $\kappa_{21}^{h}=\kappa_{12}^{h}$ and $\kappa_{21}^{r}=\kappa_{12}^{r}$, the expression can be compactified to

(12) $$\varphi_{11}=\frac{R}{2}(\kappa_{22}^{h}+\kappa_{21}^{h})+\frac{\rho \bar{\rho }}{2}(\kappa_{22}^{r}+\kappa_{21}^{r})+\frac{{\bar{\rho }}^{2}}{4}\kappa_{11}^{cc}+\frac{\rho \bar{\rho }}{4}(\kappa_{11}^{ct}+\kappa_{11}^{tc})+\frac{\rho^{2}}{4}\kappa_{11}^{tt}.$$

Example 4 *Analyzing the phase elucidates the differences between PO and H-i in Example 2. The point is that although both relationships have $\kappa_{11}=1$, the phased components are different. In the PO case, we have $\left(\kappa_{11}^{cc},\kappa_{11}^{ct},\kappa_{11}^{tc},\kappa_{11}^{tt})=(\bar{\rho },0,\rho ,0\right)$, while the same coefficients are (1,0,0,0) for H-i. Inserting these values into equation (10) produces the formulas for $\varphi_{11}$ given in (7).*

## Generalized kinship coefficients

A key idea introduced by Karigl (1981), was to express identity coefficients in terms of *generalized kinship coefficients*. Recursive formulas for such coefficients were given by Karigl and further developed by other authors, allowing efficient computation of identity coefficients.

Passing to the two-locus situation, it is natural to seek a similar generalization of two-locus kinship coefficients. Thompson (1988) used special cases of this to compute $\varphi_{11}$, but to the best of our knowledge no general treatment has been given. Indeed, this will prove to be the main ingredient in computing the two-locus coefficients *K* and *D*.

We begin by reviewing the single-locus case.

### Generalized single-locus kinship coefficients

The pairwise kinship coefficient *φ* generalizes naturally to three or more individuals. For example, Karigl (1981) used the notation $\varphi_{abc}$ to denote the probability that homologous alleles drawn from individuals *a*, b, and *c* are all IBD. We will adopt a more flexible notation, close to that of Weeks and Lange (1988), writing the above three-person coefficient as Φ([a,b,c]). A crucial idea of Weeks and Lange (1988) was to consider multiple groups of IBD alleles simultaneously. For example, the coefficient Φ([a,b],[c,d]) denotes the probability that if one allele is sampled at random from each of *a*, *b*, c, and *d*, then the alleles from *a* and *b* are IBD, and the ones from *c* and *d* are IBD, but different from those in the first group. More generally, a *generalized kinship pattern* is a finite collection of *blocks* of pedigree members,

(13) $$G=\{[a_{1},\ldots ,a_{n}],[a_{n+1},\ldots ,],\ldots [\ldots ,a_{N}]\}.$$

The associated *generalized kinship coefficient*Φ(G) is the probability that if one allele is sampled from each individual (with replacement if the individual is repeated), then the alleles within each block are all IBD, while alleles from different blocks are not IBD.

A few simple properties of generalized kinship coefficients are worth noticing. Firstly, Φ(G) is invariant under permutations of the blocks, and also under permutations of the individuals within a block. If any block of G contains two different founders, or indeed any unrelated individuals, then Φ(G)=0. Moreover, if any individual occurs in more than two blocks, this also implies Φ(G)=0.

### Generalized two-locus kinship coefficients

Let $L_{1}$ and $L_{2}$ be fixed autosomal loci with recombination rate *ρ*. If $G_{1}$ and $G_{2}$ are generalized single-locus kinship patterns, we write $G_{1}⋈G_{2}$ for the simultaneous occurrence of $G_{1}$ and $G_{2}$ at loci $L_{1}$ and $L_{2},$ respectively. The corresponding *generalized two-locus kinship coefficient* is the probability

(14) $$\Phi (G_{1}⋈G_{2})\;:\;=P(G_{1}\;\text{at}\;L_{1}\;\text{and}\;G_{2}\;\text{at}\;L_{2}).$$

As with all the previous two-locus coefficients, this probability is a function of *ρ*.

Whenever a kinship pattern $G_{1}⋈G_{2}$ involves multiple alleles from the same individual, we must specify whether or not these belong to the same gamete. To this end, we add a *segregation index* superscript to each allele (Thompson 1988; Lange and Sinsheimer 1992). Note that the indices themselves are irrelevant; only equality between indices matters, and only if attached to the same individual. For example, the previously defined two-locus kinship $\varphi_{11}^{ab}$ can be expressed as $\Phi ([a^{1},b^{1}]⋈[a^{1},b^{1}])$, but also as, e.g. $\Phi ([a^{x},b^{y}]⋈[a^{x},b^{y}])$.

Even though the segregation index carries no inherent meaning, the following convention is useful. If the involved gamete is a transmission from a parent *x* to a child *y*, we may use *y* as a segregation index and write $x^{y}$. This notation is particularly efficient in implementations and is used extensively in the recursion formulas in Appendix A. It has, however, one notable shortcoming that seems to have gone unnoticed by previous authors. If *y* is the result of selfing of *x*, there are two different gametes segregating from *x* to *y*. Since these require different labels, the notation must be augmented in this case, e.g. y1 and y2. We note that the software MORGAN, which implements the algorithm of Thompson (1988), does not support pedigrees with selfing.

A recursive algorithm for computing $\Phi (G_{1}⋈G_{2})$ for any $G_{1},G_{2}$ in any pedigree, is given in Appendix A.

## Two-locus identity coefficients

In this section, we show how the generalized two-locus kinship coefficients allow us to calculate two-locus identity coefficients. We split the presentation into three steps, in increasing order of pedigree complexity, which have different computational demands. We start with the unilineal case, where a simple trick provides efficient calculation of the matrix *K*.

### Unilineal relationships

Let R=(a,b,P) be a unilineal relationship, and K=K(ρ) its matrix of two-locus IBD coefficients. Recall from equation (3) that *K* is uniquely determined by $\kappa_{11}$.

As evident from Example 1, we cannot generally compute $\kappa_{11}$ directly from $\varphi_{11}$. Moreover, equation (10) shows why: the phased components of $\kappa_{11}$ contribute unequally to $\varphi_{11}$. However, it turns out that we can recover $\kappa_{11}$ by considering a slightly more complex, generalized kinship coefficient, constructed to balance the contributions.

Theorem 1 *For any unilineal relationship (a,b,P), the coefficient $\kappa_{11}(\rho )$ satisfies*

(15) $$\frac{{\bar{\rho }}^{2}\rho^{2}}{16}\kappa_{11}(\rho )=\Phi (H^{*}),$$

**Fig. 3.**
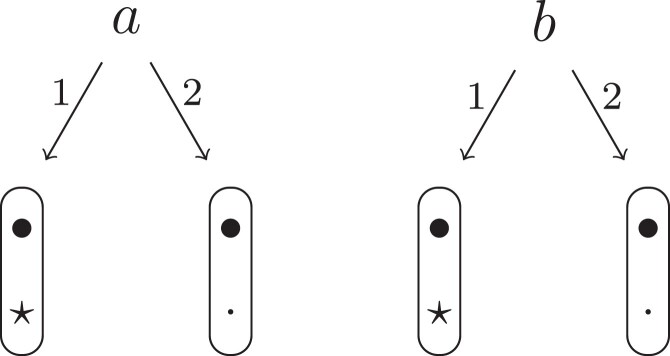
The two-locus IBD pattern $H^{*}=\{[a^{1},a^{2},b^{1},b^{2}]\}⋈\{[a^{1},b^{1}],[a^{2}],[b^{2}]\}$ used to compute $\kappa_{11}$ for unilineal relationships. Two gametes are emitted from each of *a* and *b*. At the first locus, all four alleles are IBD. At the second locus, exactly one gamete of *a* is IBD with exactly one gamete of *b*.

*where* $H^{*}=\{[a^{1},a^{2},b^{1},b^{2}]\}⋈\{[a^{1},b^{1}],[a^{2}],[b^{2}]\}$ *is the two-locus IBD pattern in* Fig. 3.

Proof The crucial point is that $H^{*}$ has the same probability under each of the four cis/trans combinations underlying $\kappa_{11}$, shown in the top row of Fig. 2. To see this, note that if the IBD alleles in *a* are *in cis*, the two gametes from *a* dictated by $H^{*}$ (cf. Fig. 3) occur with probability $\frac{1}{2}\bar{\rho }$ and $\frac{1}{2}\rho$, respectively. On the other hand, if the alleles are *in trans*, these probabilities are simply switched. Hence the total probability of *a*’s gametes is always $\frac{1}{4}\rho \bar{\rho }$. Clearly, the same holds for *b*, and it follows that $\Phi (H^{*})=(\frac{1}{4}\bar{\rho }\rho {)}^{2}(\kappa_{11}^{cc}+\kappa_{11}^{ct}+\kappa_{11}^{tc}+\kappa_{11}^{tt})=\frac{1}{16}{\bar{\rho }}^{2}\rho^{2}\kappa_{11}$ as claimed. ȃ□

Theorem 1 enables efficient calculation of $\kappa_{11}(\rho )$, and thereby the entire matrix K(ρ), for any ρ>0. The endpoint ρ=0 is trivial, as previously explained.

The four phased coefficients $\kappa_{11}^{cc},\ldots ,\kappa_{11}^{tt}$ can also be computed using a similar technique as that in Theorem 1. The idea is to find four different generalized IBD patterns $H_{1},\ldots ,H_{4}$ whose coefficients are linear expressions in $\kappa_{11}^{cc},\ldots ,\kappa_{11}^{tt}$. By choosing $H_{1},\ldots ,H_{4}$ such that the resulting system of linear equations has full rank, this can then be solved for the phased coefficients. Details are given in Appendix B.

### Bilineal relationships

Moving on to bilineal relationships, we now assume (by Lemma 1) that *a* and *b* are nonfounders of P. Let f,m denote the parents of *a*, and g,n the parents of *b*, with the understanding that some of these may coincide. To simplify matters we assume that a≠b, noting that if a=b, then *K* is trivially determined by $\kappa_{22}(\rho )=1$ for all *ρ*.

Before continuing, it is worth noting why Theorem 1 no longer holds in the bilineal case. When one or both loci may share 2 alleles IBD, the expression for $\Phi (H^{*})$ given in the proof of that theorem, gains several additional terms which obstruct an explicit solution for $\kappa_{11}$.

Our approach for computing *K*, and also the 9×9 two-locus identity matrix *D* in the next section, is inspired by the method used by Lange and Sinsheimer (1992) to calculate the single-locus coefficients $\delta_{1},\ldots ,\delta_{15}$. They noted that, for example, the detailed identity state $S_{10}^{*}$ corresponds to (in our notation) the generalized kinship pattern $\left\{[f^{a},g^{b}],[m^{a}],[n^{b}]\right\}$. Hence we have $\delta_{10}^{ab}=\Phi ([f^{a},g^{b}],[m^{a}],[n^{b}])$, which by means of a recursive algorithm enabled Lange and Sinsheimer to compute $\delta_{10}^{ab}$ in any pedigree.

In the same fashion one may define generalized IBD patterns $J_{9},\ldots ,J_{15}$ corresponding to all detailed states $S_{9}^{*},\ldots ,S_{15}^{*}$ in which *a* and *b* are noninbred (cf. Fig. 1a):

(16) $$\begin{array}{c} J_{9}\;=\;\{[f^{a},g^{b}],[m^{a},n^{b}]\},\\ J_{10}\;=\;\{[f^{a},g^{b}],[m^{a}],[n^{b}]\},\\ J_{11}\;=\;\{[m^{a},n^{b}],[f^{a}],[g^{b}]\},\\ J_{12}\;=\;\{[f^{a},n^{b}],[m^{a},g^{b}]\},\\ J_{13}\;=\;\{[f^{a},n^{b}],[m^{a}],[g^{b}]\},\\ J_{14}\;=\;\{[m^{a},g^{b}],[f^{a}],[n^{b}]\},\\ J_{15}\;=\;\{[f^{a}],[m^{a}],[g^{b}],[n^{b}]\}. \end{array}$$

(We will complete this list in the next section by including patterns corresponding to $S_{1}^{*},\ldots ,S_{8}^{*}$.)

Let $B_{0},B_{1}B_{2}$, be the partition of indices {9,…,15} according to the number of IBD alleles in each of the detailed states:

(17) $$\begin{array}{rl} B_{0} & =\{15\},\\ B_{1} & =\{10,11,13,14\},\\ B_{2} & =\{9,12\}. \end{array}$$

With these definitions the single-locus IBD coefficients could be obtained by the formula

(18) $$\kappa_{i}=\sum_{r\in B_{i}}\Phi (J_{r}),\;i=0,1,2.$$

Switching to the two-locus situation, our aim is to give a two-locus version of (18). To compute a two-locus IBD coefficient, say $\kappa_{00}$, we might proceed as follows: $\kappa_{00}$ is the probability that *a* and *b* share 0 alleles IBD at both loci, i.e. that both loci are in state $S_{15}^{*}$. In other words, $\kappa_{00}=\Phi (J_{15}⋈J_{15})$. Similarly, $\kappa_{20}$ is the probability that the state at $L_{1}$ is either $S_{9}^{*}$ or $S_{12}^{*}$ (the states where *a* and *b* share 2 alleles), while $L_{2}$ is in state $S_{15}^{*}$, thus $\kappa_{20}=\Phi (J_{9}⋈J_{15})+\Phi (J_{12}⋈J_{15})$. By this reasoning, we obtain the following general result:

Theorem 2 *Suppose *a* and *b* are nonfounders in P, and let $J_{9},\ldots ,J_{15}$ be the IBD patterns defined in (16). The two-locus IBD coefficients $\kappa_{ij}$, for i,j=0,1,2 are then given by.*

(19) $$\kappa_{ij}=\sum_{r\in B_{i},s\in B_{j}}\Phi (J_{r}⋈J_{s}).$$

A complete overview of the detailed two-locus states and their corresponding patterns $J_{r}⋈J_{s}$, is given in Table 1. This table also shows how to obtain the phased versions by further partitioning the sums given in Theorem 2. For example, equation (19) dictates that

(20) $$\kappa_{22}=\Phi (J_{9}⋈J_{9})+\Phi (J_{9}⋈J_{12})+\Phi (J_{12}⋈J_{9})+\Phi (J_{12}⋈J_{12}).$$

Inspecting the corresponding states (top two rows of Table 1) it is evident that the first and fourth terms of (20) contribute to $\kappa_{22}^{h}$, while the other two belong to $\kappa_{22}^{r}$.

**Table 1.** Formulas for the phased two-locus IBD coefficients in bilineal relationships.

Detailed two-locus IBD states				Formula
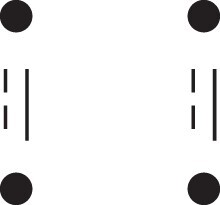	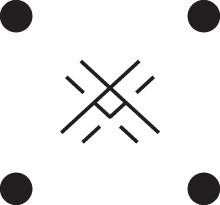			$\kappa_{22}^{h}=\Phi (J_{9}⋈J_{9})+\Phi (J_{12}⋈J_{12})$
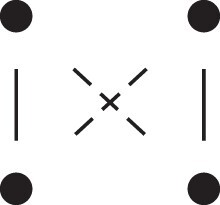	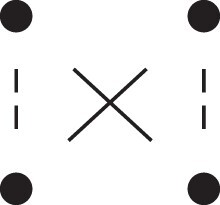			$\kappa_{22}^{r}=2\cdot \Phi (J_{9}⋈J_{12})$
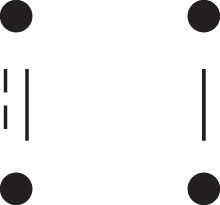	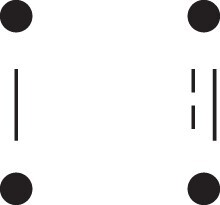	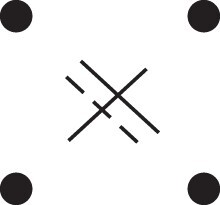	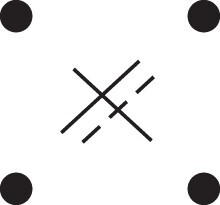	κ21h=Φ(J9⋈J10)+Φ(J9⋈J11)1111+Φ(J12⋈J13)+Φ(J12⋈J14)
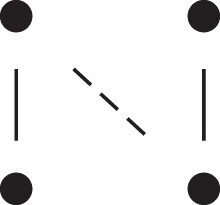	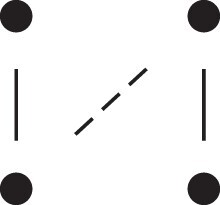	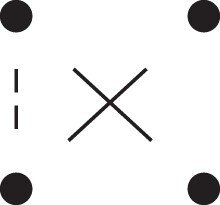	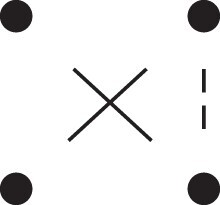	κ21r=Φ(J9⋈J13)+Φ(J9⋈J14)1111+Φ(J12⋈J10)+Φ(J12⋈J11)
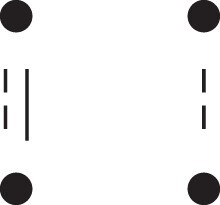	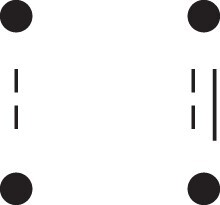	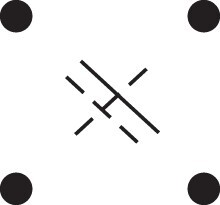	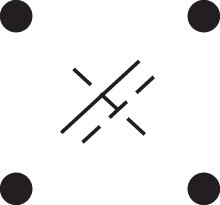	$\kappa_{12}^{h}=\kappa_{21}^{h}$
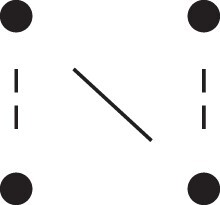	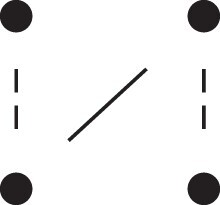	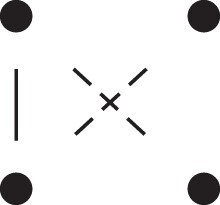	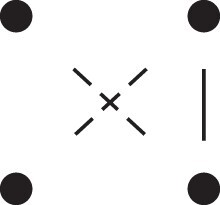	$\kappa_{12}^{r}=\kappa_{21}^{r}$
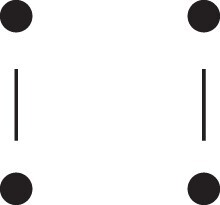	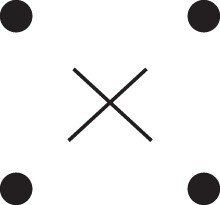			$\kappa_{20}=\Phi (J_{9}⋈J_{15})+\Phi (J_{12}⋈J_{15})$
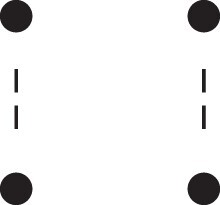	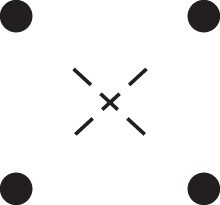			$\kappa_{02}=\kappa_{20}$
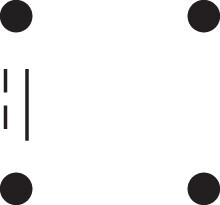	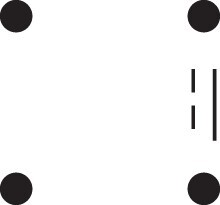	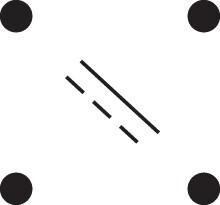	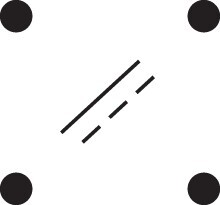	κ11cc=Φ(J10⋈J10)+Φ(J11⋈J11)1111+Φ(J13⋈J13)+Φ(J14⋈J14)
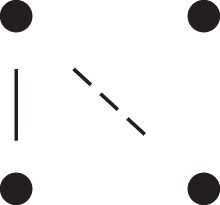	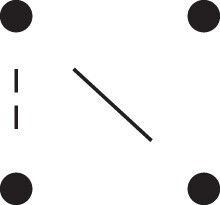	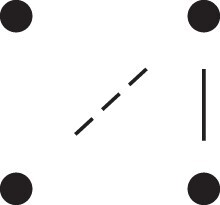	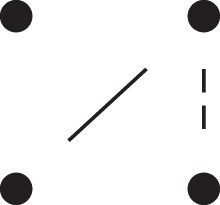	$\kappa_{11}^{ct}=2\cdot \Phi (J_{10}⋈J_{13})+2\cdot \Phi (J_{11}⋈J_{14})$
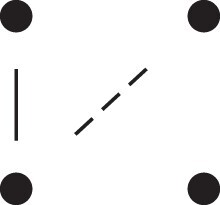	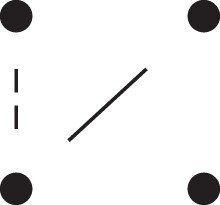	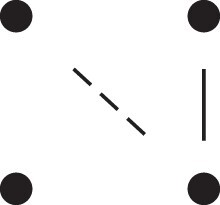	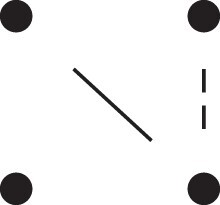	$\kappa_{11}^{tc}=2\cdot \Phi (J_{10}⋈J_{14})+2\cdot \Phi (J_{11}⋈J_{13})$
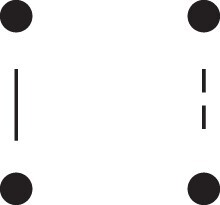	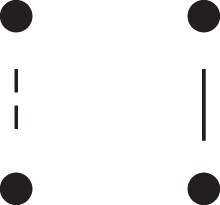	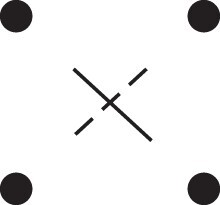	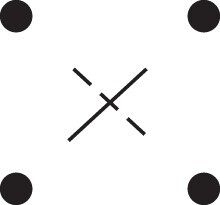	$\kappa_{11}^{tt}=2\cdot \Phi (J_{10}⋈J_{11})+2\cdot \Phi (J_{13}⋈J_{14})$
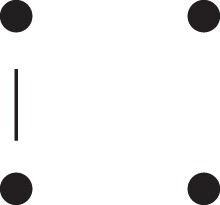	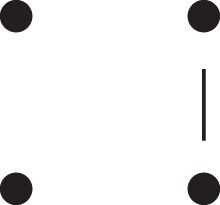	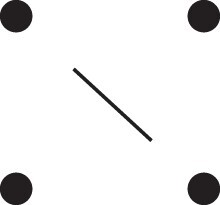	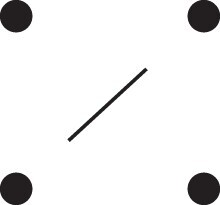	κ10=Φ(J10⋈J15)+Φ(J11⋈J15)1111+Φ(J13⋈J15)+Φ(J14⋈J15)
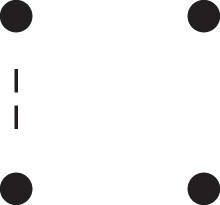	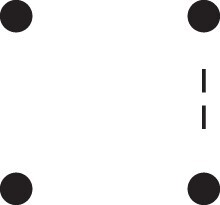	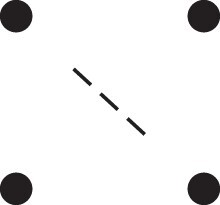	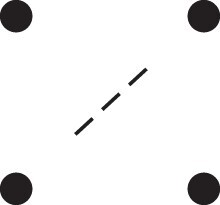	$\kappa_{01}=\kappa_{10}$
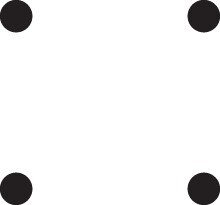				$\;\kappa_{00}=\Phi (J_{15}⋈J_{15})$

To the left of each formula are shown the corresponding detailed IBD states, where solid (resp. dashed) lines indicate IBD at the first (resp., second) locus. The diagrams otherwise follow the conventions of Fig. 1a. Definitions of the single-locus patterns $J_{9},\ldots ,J_{15}$ are given in the main text.

### General relationships

The method of the previous section generalizes immediately to the full-blown matrix *D* of 81 two-locus identity coefficients between *a* and *b*, which we now allow to be inbred. The main challenge is the volume of cases, as there are now 15⋅15=225 detailed two-locus states to consider, each corresponding to a generalized two-locus kinship coefficient of the form $\Phi (J_{r}\;⋈\;J_{s})$, r,s=1,…,15.

First of all, we complete the list (16) by adding the patterns corresponding to the states $S_{1}^{*},\ldots ,S_{8}^{*}$ for inbred *a* and/or *b*:

(21) $$\begin{array}{c} J_{1}=\{[f^{a},m^{a},g^{b},n^{b}]\},\\ J_{2}=\{[f^{a},m^{a},g^{b}],[n^{b}]\},\\ J_{3}=\{[f^{a},m^{a},n^{b}],[g^{b}]\},\\ J_{4}=\{[f^{a},g^{b},n^{b}],[m^{a}]\},\\ J_{5}=\{[f^{a}],[m^{a},g^{b},n^{b}]]\},\\ J_{6}=\{[f^{a},m^{a}],[g^{b},n^{b}]\},\\ J_{7}=\{[f^{a},m^{a}],[g^{b}],[n^{b}]\},\\ J_{8}=\{[f^{a}],[m^{a}],[g^{b},n^{b}]\}. \end{array}$$

In the same manner as (17), we define subsets $C_{1},\ldots ,C_{9}\subseteq \{1,\ldots ,15\}$ as the indices corresponding to the condensed states, i.e. so that $\Delta_{i}=\sum_{r\in C_{i}}\delta_{r}$.

(22) $$\begin{array}{c} C_{1}=\{1\},\;\;\;\;\;\;\\ C_{2}=\{6\},\;\;\;\;\;\;\\ C_{3}=\{2,3\},\;\;\;\;\;\\ C_{4}=\{7\},\;\;\;\;\;\;\\ C_{5}=\{4,5\},\;\;\;\;\;\\ C_{6}=\{8\},\;\;\;\;\;\;\;\\ C_{7}=\{9,12\},\;\;\;\;\;\\ C_{8}=\{10,11,13,14\},\\ C_{9}=\{15\}.\;\;\;\;\; \end{array}$$

We can then give the most general result of this paper, providing an implementation-friendly formula for the 81 two-locus identity coefficients of any pairwise relationship (a,b,P).

Theorem 3 *For any nonfounders a,b∈P their two-locus condensed identity coefficients $\Delta_{ij}$, i,j∈1,…,9 are given by*

(23) $$\Delta_{ij}=\sum_{r\in C_{i},s\in C_{j}}\Phi (J_{r}⋈J_{s}),$$

where $J_{1},\ldots ,J_{15}$ are the generalized IBD patterns defined in (16) and (21).

The assumption that a,b are nonfounders can easily be circumvented by extending the pedigree before applying the theorem. For example, if *a* is a founder of P, let $P^{\prime }$ be the pedigree resulting from adding both of *a*’s parents, as unrelated founders. Theorem 3 can then be applied to $\left(a,b,P^{\prime }\right)$.

## Implementation

The algorithms described in this paper are implemented in the R package ribd (Vigeland 2020), which is part of the ped suite collection of packages for pedigree analysis in R (Vigeland 2021). Detailed explanations and many examples are included in the documentation of the functions twoLocusKinship, twoLocusIBD and twoLocusIdentity.

In light of the extensive recursions needed to calculate generalized two-locus kinship coefficients, care should be taken to alleviate the computational burden. A naive application of Theorem 3 requires the calculation of $15^{2}=225$ generalized two-locus kinship coefficients $\Phi (J_{r}⋈J_{s})$ to obtain the complete matrix *D*. However, this number can be almost halved by exploiting linear dependencies. Let $n_{ij}=|C_{i}|\cdot |C_{j}|$ be the number of terms in equation (23), i.e.

(24) $$(n_{ij}{)}_{i,j\in \{1,\ldots ,9\}}=\left(\begin{array}{ccccccccc} 1 & 1 & 2 & 1 & 2 & 1 & 2 & \;4 & 1\\ 1 & 1 & 2 & 1 & 2 & 1 & 2 & \;4 & 1\\ 2 & 2 & 4 & 2 & 4 & 2 & 4 & \;8 & 2\\ 1 & 1 & 2 & 1 & 2 & 1 & 2 & \;4 & 1\\ 2 & 2 & 4 & 2 & 4 & 2 & 4 & \;8 & 2\\ 1 & 1 & 2 & 1 & 2 & 1 & 2 & \;4 & 1\\ 2 & 2 & 4 & 2 & 4 & 2 & 4 & \;8 & 2\\ \;4 & \;4 & \;8 & \;4 & \;8 & \;4 & \;8 & \;16 & \;4\\ 1 & 1 & 2 & 1 & 2 & 1 & 2 & \;4 & 1 \end{array}\right).$$

Since the coefficients in row 8 and column 8 are the most expensive (numbers shown in bold), it is most profitable to obtain these by other means. First, using the rows sums of *D*, we have $\Delta_{i8}=\Delta_{8}-\sum_{\;j\neq 8}\Delta_{ij}$, for each i≠8. Then, by the columns sums, $\Delta_{8j}=\Delta_{8}-\sum_{i\neq 8}\Delta_{ij}$, for all *j* (including 8). This procedure eliminates 104 of the 225 terms, leaving only 121 (≈53%) generalized coefficients requiring recursive calculation.

For unilineal relationships, the computation of two-locus IBD coefficients can be performed very quickly using Theorem 1. On a standard laptop computer (Intel core i5 CPU @ 1.60GHz, 16 Gb RAM, Windows 10, 64-bit R), ribd computes $\kappa_{11}$ in 0.01 s for 5th cousins, and in 0.1 s for 50th cousins. Bilinear and, in particular, inbred relationships are more computer intensive, even with the trick described in the preceding paragraph. For example, the current implementation takes 0.5 s to compute the complete 9×9 matrix *D* for a pair of siblings resulting from brother-sister mating, and about 30 s after 5 generations of brother-sister mating.

A particular feature of the ped suite is the support of *founder inbreeding*, i.e. the assignment of nonzero inbreeding coefficients to any pedigree founders. As shown in Vigeland (2020, Section 6.2), such founder inbreeding generally leads to ill-defined multilocus IBD coefficients, except in cases of *complete* inbreeding. All two-locus functions in ribd support completely inbred founders (and give an error if encountering partially inbred founders). For example, the H-i pedigree featured in Example 2 can be analyzed as follows, after loading the ribd package in R:

# Half siblings with completely inbred parent

x = halfSibPed()

founderInbreeding(x, ids = 2) = 1

# A two-locus kinship coefficient

twoLocusKinship(x, ids = 4:5, rho = 0.25)

[1] 0.140625

# The two-locus IBD matrix (same for any rho)

twoLocusIBD(x, ids = 4:5, rho = 0.25)

The output of the last command (not shown) is the matrix K in equation (6).

If either of the two individuals, say *a*, is a founder, then the algorithm described in Section “General relationships” requires that we extend the pedigree by adding the parents of *a* before applying Theorem 3. However, this cannot be done adequately if *a* is completely inbred; hence in this particular case founder inbreeding is not supported in the current implementation.

In order to check the implementation, numerical validation was performed against a wide variety of previously published two-locus probability formulas. Details and source code for these efforts can be found in the documentation of ribd, including examples from Weir and Cockerham (1969), Denniston (1975), Donnelly (1983), Thompson (1988), Bishop and Williamson (1990), Almasy and Blangero (1998), Egeland and Slooten (2016), and Vigeland (2021).

Of particular interest is the work of Almasy and Blangero (1998) (AB98 in the following), which provides extensive tables of formulas for $\kappa_{11}$ in unilineal relationships, and IBD correlation formulas (which are simple functions of the $\kappa_{ij}$’s) for many bilineal cases. Some mistakes in these formulas were discovered as a result of the comparison. Given the high impact of AB98 we briefly record these here: In Table 4 of AB98, the correlation coefficient of “Double second cousins (type A)” should be $1-\frac{48}{7}\theta +\frac{134}{7}\theta^{2}-\frac{200}{7}\theta^{3}+\frac{172}{7}\theta^{4}-\frac{80}{7}\theta^{5}+\frac{16}{7}\theta^{6}$ (they use *θ* to denote the recombination fraction). Furthermore, Table 6 has a misprint in the entry for “First cousin and second cousin,” where the coefficient of $\theta^{6}$ should be 8,240, not 8,420.

## Discussion

Considering the enduring interest in two-locus IBD probabilities, the lack of general algorithms may seem surprising. One explanation may be that for simple relationships explicit formulas can be obtained by direct calculation. Below we briefly discuss two applications of two-locus coefficients which, with the methods of the present paper, can now be extended to larger classes of pedigrees.

In forensic kinship testing, a hypothesized relationship R between two individuals is typically tested by evaluating the likelihood ratio LR=P(G∣R)/P(G∣unrelated), where *G* denotes the genotypes at a predetermined set of markers. Egeland and Slooten discovered that if *G* is interpreted as a random variable, the expectation E[LR] is independent of allele frequencies, and can be expressed by a remarkably simple formula (Egeland and Slooten 2016). In the case of two markers, let $M^{\left(1\right)}$ be the matrix

(25) $$M^{\left(1\right)}=\left(\begin{array}{ccc} 1 & 1 & 1\\ 1 & \frac{n_{1}+3}{4} & \frac{n_{1}+1}{2}\\ 1 & \frac{n_{1}+1}{2} & \frac{n_{1}(n_{1}+1)}{2} \end{array}\right),$$

where $n_{1}$ is the number of alleles at the first marker, and define $M^{\left(2\right)}$ similarly for the second marker. For convenience, we use 0-indexing for the entries of these matrices, e.g. $M_{11}^{\left(1\right)}=(n_{1}+3)/4$. Now, suppose $\left(\kappa_{ij}(\rho )\right)$ are the two-locus IBD coefficients of R, where *ρ* is the recombination fraction between the markers. Furthermore, suppose that $\left(\kappa_{ij}^{\prime }(\rho )\right)$ are the coefficients of the *true* relationship $R^{\prime }$, which may be different from R. (We assume that both R and $R^{\prime }$ are noninbred.) The expected likelihood ratio is then the following sum (Egeland and Slooten 2016, eq. 2.18),

(26) $$E[LR]=\sum_{i,j,i^{\prime },j^{\prime }\in \{0,1,2\}}\kappa_{ij}\kappa_{i^{\prime }j^{\prime }}^{\prime }M_{ii^{\prime }}^{\left(1\right)}M_{\;jj^{\prime }}^{\left(2\right)}.$$

**Fig. 4.**
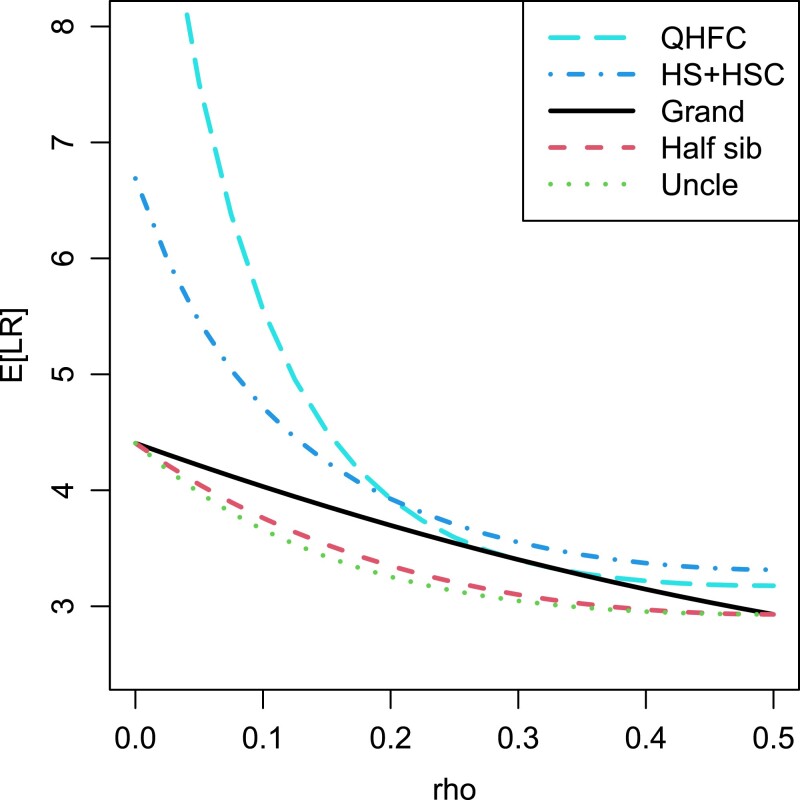
Expected LR with two markers with 10 and 15 alleles, for various relationships. QHFC = Quadruple half-first cousins. HS + HSC = Simultaneous half-siblings and half-second cousins.

In Fig. 4, we reproduce and expand a result of Egeland and Slooten (2016, Example 3.4). Here, E[LR] is shown as a function of *ρ* for various relationships (assuming $R^{\prime }=R$) with two markers of 10 and 15 equally frequent alleles. The three lowest curves, corresponding to grandparent–grandchild, half-siblings and uncle-nephew relationships, agree with the lower part of Fig. 2 in Egeland and Slooten (2016). Two further relationships have been added, namely quadruple half-first cousins (QHFC) and simultaneous half-siblings and half-second cousins (HS+HSC). These were chosen because they are genetically close to the first three, and also to illustrate the utility of the algorithm presented in this paper, since exact formulas for $\kappa_{ij}(\rho )$ are not available (and nontrivial to work out) for these relationships. An interesting observation from Fig. 4 is that the ranking of the relationships according to E[LR] depends on the distance between the markers.

Another powerful application of two-locus coefficients is in the study of realized relatedness, i.e. the actual IBD segments shared by a pair of related individuals. Guo (1995, 1996) showed that for noninbred individuals, the variance in proportion of genome-shared IBD can be expressed as a double integral involving two-locus IBD probabilities, and used this to compute the said variance in special cases. The same technique can be used to compute other similar variances. For instance, for a given pair of noninbred relatives, let $\left(k_{0},k_{1},k_{2}\right)$ be the actual proportions of their genomes where they share 0,1,2 alleles IBD, respectively. For each j=0,1,2 we have $E(k_{j})=\kappa_{j}$. For a chromosome of length *L*, we can write $k_{j}=(1/L)\int_{0}^{L}I(K_{x}=j)\;dx$, where I(⋅) is an indicator variable and $K_{x}$ is the number of IBD alleles (0, 1 or 2) at locus *x*. The variance formula $\mathrm{Var}\;(Y)=E(Y^{2})-E{\left(Y\right)}^{2}$ then gives:

(27) $$\begin{array}{rl} \mathrm{Var}\;(k_{j}) & =E\left[{\left(\frac{1}{L}\int_{0}^{L}I(K_{x}=j)\;dx\right)}^{2}\right]-E{\left[\frac{1}{L}\int_{0}^{L}I(K_{x}=j)\;dx\right]}^{2}\\ & =\frac{1}{L^{2}}\int_{0}^{L}\int_{0}^{L}I(K_{x}=K_{y}=j)\;dy\;dx-\kappa_{j}^{2}\\ & =\frac{1}{L^{2}}\int_{0}^{L}\int_{0}^{L}\kappa_{\;jj}(\rho_{xy})\;dy\;dx-\kappa_{j}^{2}. \end{array}$$

Here, $\rho_{xy}$ is the recombination rate between loci *x* and *y*. Assuming a Poisson crossover process, Haldane’s map function entails $\rho_{xy}=\frac{1}{2}-\frac{1}{2}e^{-2d(x,y)}$, where d(x,y) is the genetic distance (in Morgan) between *x* and *y*. Hill and Weir (2011) used an approach resembling (27) to compute the variances of $k_{j}$ and other measures of realized relatedness. However, only special cases of noninbred relationships were considered, in which the required two-locus IBD probabilities could be obtained by direct, but tedious calculations. In contrast, the algorithm and implementation presented here allow these variances to be also obtained in complex pedigrees. This includes inbred relationships, where variances in the realized proportions in states $S_{1},\ldots ,S_{9}$ may be defined analogously to (27). Further details and examples, including numerical validations of Hill and Weir (2011) are given in the documentation of the ribd package.

### Conclusion

This paper presents an algorithm for computing joint IBD probabilities at two linked loci, called two-locus identity coefficients, in any pedigree. Previous work in this area have focused on simple cases of noninbred relationships, where explicit formulas can be obtained by brute force. In contrast, the method described here applies to any pairwise relationship, both noninbred and inbred. The inbred case requires as many as 81 two-locus coefficients, which may seem like a daunting task. However, they can all be expressed in terms of generalized two-locus kinship coefficients, for which a recursive algorithm is given. All methods, including numerous examples, are implemented in the R package ribd, which runs on all common platforms and is freely available from CRAN. As a result, a variety of methods and applications, previously restricted to simple, special cases, may now be applied and explored in general pedigrees.

## Data Availability

All source code is available on GitHub at https://github.com/magnusdv/ribd.

## Appendix A: A recursive algorithm for generalized two-locus kinship coefficients

We will here describe a recursive algorithm for computing any generalized two-locus kinship coefficient, including the terms $\Phi (J_{r}⋈J_{s})$ used in Theorems 2 and 3 to obtain two-locus identity coefficients. The algorithm is similar in spirit to that of Weeks and Lange (1988) (referred to as WL88 below) and Lange and Sinsheimer (1992), although some additional care is required to account for linkage between the loci.

To compute the probability of any two-locus IBD pattern H, we start by writing it in the form

(A1) $$\begin{array}{rl} H & =\{[a^{i_{1}},\ldots ,a^{i_{r}},\ldots ],[a^{\;j_{1}},\ldots ,a^{\;j_{s}},\ldots ],\ldots \}\\ & ⋈\{[a^{k_{1}},\ldots ,a^{k_{t}},\ldots ],[a^{l_{1}},\ldots ,a^{l_{u}},\ldots ],\ldots \}\\ & =\{[a^{T_{1}},\ldots ],[a^{T_{2}},\ldots ],\ldots \}⋈\{[a^{T_{3}},\ldots ],[a^{T_{4}},\ldots ],\ldots \}, \end{array}$$

where *a* is not an ancestor of any individual present in H, and the *target sets*$T_{1},\ldots T_{4}$ (some of which may be empty) contain the segregation indices of the alleles emitted from *a* in each block:

(A2) $$\begin{array}{c} T_{1}=\{i_{1},\ldots ,i_{r}\},\\ T_{2}=\{j_{1},\ldots ,j_{s}\},\\ T_{3}=\{k_{1},\ldots ,k_{t}\},\\ T_{4}=\{l_{1},\ldots ,l_{u}\}. \end{array}$$

The assumption that *a* is present in at most two blocks at each locus follows from diploidy; clearly, Φ(H)=0 otherwise. By permuting blocks and loci if necessary, we may assume w.l.o.g. that r≥s, that t≥u, and that the first locus has at least as many nonempty target sets as the second. Calling the $T_{i}$’s *sets* is justified since duplicates within any block of H can be removed without changing Φ(H). On the other hand, duplicates *across* blocks at the same locus gives Φ(H)=0, since the same gamete cannot have multiple alleles at the same locus. Thus, we may assume $T_{1}\cap T_{2}=T_{3}\cap T_{4}=\emptyset$.

The following integers are used in the recursion formulas:

(A3) $$\begin{array}{c} \nu =\;\#\{T_{1}\cup T_{2}\cup T_{3}\cup T_{4}\},\\ \;\mu =\;\#\{(T_{1}\cap T_{3})\cup (T_{2}\cap T_{4})\},\\ \sigma =\;\#\{(T_{1}\cap T_{4})\cup (T_{2}\cap T_{3})\}. \end{array}$$

Note that *ν* is the total number of distinct gametes emitted from *a*, of which μ+σ contain specified alleles from *a* at both loci. Importantly, both *ν* and the (unordered) set {μ,σ} are invariant under permutations of blocks and loci of H.

### Recursion formulas

The recursion proceeds upwards through the pedigree, in each step replacing a child *a* with its parents *f* and *m*, until only founders are left. For notational convenience, we denote parent-to-child transmissions using the child’s label as segregation index, as in $f^{a}$. As mentioned previously, this does not work with selfing, but such cases can be easily handled in the implementation.

Case 1:

r>0 , s=t=u=0
In this case, *a* is present only in the first block of the first locus. This is covered by Recursion rules 1 and 2 of WL88, giving

(A4) $$\begin{array}{rl} \Phi (\{[a^{T_{1}},\ldots ],\ldots \}⋈\{\ldots \}) & =A_{1}\cdot \Phi (\{[f^{a},\ldots ],\ldots \}⋈\{\ldots \})\\ & \;+A_{1}\cdot \Phi (\{[m^{a},\ldots ],\ldots \}⋈\{\ldots \})\\ & \;+B_{1}\cdot \Phi (\{[f^{a},m^{a},\ldots ],\ldots \}⋈\{\ldots \}), \end{array}$$

where $A_{1}=\frac{1}{2^{r}}$, and $B_{1}=1-\frac{1}{2^{r-1}}$ if s>1, and 0 otherwise.

Case 2:

r,s>0 , t=u=0
Again, *a* is present only in the first locus, and we may apply Recurrence rule 3 of WL88:

(A5) $$\begin{array}{rl} \Phi (\{[a^{T_{1}},\ldots ],[a^{T_{2}},\ldots ],\ldots \}⋈\{\ldots \}) & =A_{2}\cdot \Phi (\{[f^{a},\ldots ],[m^{a},\ldots ],\ldots \}⋈\{\ldots \})\\ & \;+A_{2}\cdot \Phi (\{[m^{a},\ldots ],[f^{a},\ldots ],\ldots \}⋈\{\ldots \}), \end{array}$$

where $A_{2}=\frac{1}{2^{r+s}}$.

Case 3:

r,t>0 , s=u=0
This is the most complicated case, with up to 9-fold recursion:

(A6) $$\begin{array}{rl} & \Phi (\{[a^{T_{1}},\ldots ],\ldots \}⋈\{[a^{T_{3}},\ldots ],\ldots \})=A_{3}\cdot \Phi (\{[f^{a},\ldots ],\ldots \}⋈\{[f^{a},\ldots ],\ldots \})\\ & \;+A_{3}\cdot \Phi (\{[m^{a},\ldots ],\ldots \}⋈\{[m^{a},\ldots ],\ldots \})+B_{3}\cdot \Phi (\{[f^{a},\ldots ],\ldots \}⋈\{[m^{a},\ldots ],\ldots \})\\ & \;+B_{3}\cdot \Phi (\{[m^{a},\ldots ],\ldots \}⋈\{[f^{a},\ldots ],\ldots \})+C_{3}\cdot \Phi (\{[f^{a},m^{a},\ldots ],\ldots \}⋈\{[f^{a},\ldots ],\ldots \})\\ & \;+C_{3}\cdot \Phi (\{[f^{a},m^{a},\ldots ],\ldots \}⋈\{[m^{a},\ldots ],\ldots \})+D_{3}\cdot \Phi (\{[f^{a},\ldots ],\ldots \}⋈\{[f^{a},m^{a},\ldots ],\ldots \})\\ & \;+D_{3}\cdot \Phi (\{[m^{a},\ldots ],\ldots \}⋈\{[f^{a},m^{a},\ldots ],\ldots \})+E_{3}\cdot \Phi (\{[f^{a},m^{a},\ldots ],\ldots \}⋈\{[f^{a},m^{a},\ldots ],\ldots \}). \end{array}$$

The coefficients are as follows, where $R_{\mu }\;:\;=\rho^{\mu }+{\bar{\rho }}^{\mu }$. (Note that $\mu =\#\{T_{1}\cap T_{3}\}$ and σ=0.)

(A7) $$\begin{array}{rl} A_{3} & =\frac{1}{2^{\nu }}{\bar{\rho }}^{\mu },\\ B_{3} & =\frac{1}{2^{\nu }}\rho^{\mu },\\ C_{3} & =\frac{1}{2^{t}}-\frac{1}{2^{\nu }}R_{\mu }\;\text{if }r>1,\text{ otherwise }0,\\ D_{3} & =\frac{1}{2^{r}}-\frac{1}{2^{\nu }}R_{\mu }\;\text{if }t>1,\text{ otherwise }0,\\ E_{3} & =1-\frac{1}{2^{t-1}}-\frac{1}{2^{r-1}}+\frac{1}{2^{\nu -1}}R_{\mu }\;\text{if both }r,t>1,\text{ otherwise }0. \end{array}$$

The first two terms of (A6) cover the possibilities where the alleles $a^{T_{1}}\cup a^{T_{3}}$ emitted from *a*, all originate from the father *f*, or all from the mother *m*. The next two terms consider the situations when all of $a^{T_{1}}$ come from *f* and all of $a^{T_{3}}$ from *m*, or *vice versa*. The $C_{3}$ terms account for the cases where the set $a^{T_{1}}$ includes alleles from both *f* and *m* (implying that *a* is inbred), while the alleles in $a^{T_{3}}$ originate either all from *f* or all from *m*. The $D_{3}$ terms are similar, but with the loci switched. The last term covers the remaining cases when both sets $a^{T_{1}}$ and $a^{T_{3}}$ contain alleles from both *f* and *m*.

Case 4:

r,s,t>0 , u=0
Here, we have

(A8) $$\begin{array}{rl} & \Phi (\{[a^{T_{1}},\ldots ],[a^{T_{2}},\ldots ],\ldots \}⋈\{[a^{T_{3}},\ldots ],\ldots \})\\ & \;=A_{4}\cdot \Phi (\{[f^{a},\ldots ],[m^{a},\ldots ],\ldots \}⋈\{[f^{a},\ldots ],\ldots \})\\ & \;+A_{4}\cdot \Phi (\{[m^{a},\ldots ],[f^{a},\ldots ],\ldots \}⋈\{[m^{a},\ldots ],\ldots \})\\ & \;+B_{4}\cdot \Phi (\{[f^{a},\ldots ],[m^{a},\ldots ],\ldots \}⋈\{[m^{a},\ldots ],\ldots \})\\ & \;+B_{4}\cdot \Phi (\{[m^{a},\ldots ],[f^{a},\ldots ],\ldots \}⋈\{[f^{a},\ldots ],\ldots \})\\ & \;+C_{4}\cdot \Phi (\{[f^{a},\ldots ],[m^{a},\ldots ],\ldots \}⋈\{[f^{a},m^{a},\ldots ],\ldots \})\\ & \;+C_{4}\cdot \Phi (\{[m^{a},\ldots ],[f^{a},\ldots ],\ldots \}⋈\{[f^{a},m^{a},\ldots ],\ldots \}), \end{array}$$

where, as before, the coefficients are found by considering the origins (*f* or *m*) of the alleles:

(A9) $$\begin{array}{cc} & A_{4}=\frac{1}{2^{\nu }}{\bar{\rho }}^{\mu }\rho^{\sigma },\\ & B_{4}=\frac{1}{2^{\nu }}{\bar{\rho }}^{\sigma }\rho^{\mu },\\ & C_{4}=\frac{1}{2^{r+s}}-\frac{1}{2^{\nu }}({\bar{\rho }}^{\mu }\rho^{\sigma }+{\bar{\rho }}^{\sigma }\rho^{\mu })\;\text{if }t>1,\text{ otherwise }0. \end{array}$$

Case 5:

r,s,t,u>0

In the final case, we have the following recursion formula:

(A10) $$\begin{array}{rl} & \Phi (\{[a^{T_{1}},\ldots ],[a^{T_{2}},\ldots ],\ldots \}⋈\{[a^{T_{3}},\ldots ],[a^{T_{4}},\ldots ],\ldots \})\\ & \;=A_{5}\cdot \Phi (\{[f^{a},\ldots ],[m^{a},\ldots ],\ldots \}⋈\{[f^{a},\ldots ],[m^{a},\ldots ],\ldots \})\\ & \;+A_{5}\cdot \Phi (\{[m^{a},\ldots ],[f^{a},\ldots ],\ldots \}⋈\{[m^{a},\ldots ],[f^{a},\ldots ],\ldots \})\\ & \;+B_{5}\cdot \Phi (\{[f^{a},\ldots ],[m^{a},\ldots ],\ldots \}⋈\{[m^{a},\ldots ],[f^{a},\ldots ],\ldots \})\\ & \;+B_{5}\cdot \Phi (\{[m^{a},\ldots ],[f^{a},\ldots ],\ldots \}⋈\{[f^{a},\ldots ],[m^{a},\ldots ],\ldots \}), \end{array}$$

where $A_{5}=\frac{1}{2^{\nu }}\rho^{\mu }{\bar{\rho }}^{\sigma }$ and $B_{5}=\frac{1}{2^{\nu }}\rho^{\sigma }{\bar{\rho }}^{\mu }$.

### Boundary formulas

Since each step replaces a child with its parents, the recursion will eventually reach a pattern $H=G_{1}⋈G_{2}$ involving only founders. The following boundary conditions, adapted from WL88, then apply to each locus $G_{i}$ individually:

Boundary condition 1 If a founder (or in fact any person) occurs in more than two blocks of *$G_{i}$*, then *Φ(H)=0*.

Boundary condition 2 If two different founders occur in the same block of $G_{i}$, then Φ(H)=0.

Finally, we have a single boundary rule where $G_{1}$ and $G_{2}$ are considered together.

Boundary condition 3 If all individuals in *H* are founders, and none of the above boundary rules applies, then *$\Phi (H)=\prod_{a}B_{a}$*where the product is over all distinct founders appearing in *H*, and the factors *$B_{a}$*are computed as follows. After a suitable permutation of the blocks, we can write $H=\{[a^{T_{1}}],[a^{T_{2}}],\ldots \}⋈\{[a^{T_{3}}],[a^{T_{4}}],\ldots \}$, where each block of H involves the alleles of a single founder (otherwise Boundary condition 2 would apply). With *ν*, *μ* and *σ* as in (A3), we claim that the *ν* gametes emitted from *a* contain either exactly *μ* nonrecombinants and *σ* recombinants, or *vice versa*. Indeed, this follows from the observation that, since *a* is noninbred, the gametes corresponding to $T_{1}\cap T_{3}$ and $T_{2}\cap T_{4}$ are either all nonrecombinant or all recombinant, and similarly, but oppositely, for $T_{1}\cap T_{4}$ and $T_{2}\cap T_{3}$. Hence, the contribution of *a* to Φ(H) is

(A11) $$B_{a}=\frac{1}{2^{\nu }}({\bar{\rho }}^{\mu }\rho^{\sigma }+{\bar{\rho }}^{\sigma }\rho^{\mu }).$$

Note that if $T_{3}$ and $T_{4}$ are empty, i.e. if *a* occurs only in $G_{1}$, then μ=σ=0 reduces $B_{a}$ to $1/2^{\nu -1}$. From this, it follows that if $G_{2}$ is empty, then $\Phi (H)=\Phi (G_{1})=1/2^{m_{1}-m_{2}}$, where $m_{1}$ is the total number of gametes and $m_{2}$ the number of distinct individuals, in agreement with Boundary condition 3 of WL88.

## Appendix B: Phased two-locus coefficients of unilineal relationships

The purpose of this appendix is to derive a formula for the phased coefficients $\kappa_{11}^{cc}$, $\kappa_{11}^{ct}$, $\kappa_{11}^{tc}$ and $\kappa_{11}^{tt}$ in the unilineal case. Recall that in this case we cannot assume that *a* and *b* are nonfounders, so we cannot rely on the generalized patterns $J_{1},\ldots ,J_{15}$.

The approach is similar to the computation of $\kappa_{11}$ in Theorem 1. We start off by defining the following generalized two-locus IBD patterns:

(B1) $$\begin{array}{c} H_{1}=\{[a^{1},b^{1}]\}⋈\{[a^{1},b^{1}]\},\\ H_{2}=\{[a^{1},b^{1}],[b^{2}]\}⋈\{[a^{1},b^{2}],[b^{1}]\},\\ H_{3}=\{[a^{1},b^{1}],[a^{2}]\}⋈\{[a^{2},b^{1}],[a^{1}]\},\\ H_{4}=\{[a^{1},b^{1}],[a^{2}],[b^{2}]\}⋈\{[a^{2},b^{2}],[a^{1}],[b^{1}]\}. \end{array}$$

Theorem 4
*For a unilineal relationship (a,b,P), the phased coefficients $\kappa_{11}^{cc},\ldots ,\kappa_{11}^{tt}$ are determined as follows:*

- *For ρ≠0.5, we have*
  (B2)
  $$\begin{array}{r} \left(\begin{array}{c} \kappa_{11}^{cc}\\ \kappa_{11}^{ct}\\ \kappa_{11}^{tc}\\ \kappa_{11}^{tt} \end{array}\right)=\frac{1}{({\bar{\rho }}^{3}-\rho^{3}{)}^{2}}\left(\begin{array}{cccc} {\bar{\rho }}^{4} & -{\bar{\rho }}^{2}\rho & -{\bar{\rho }}^{2}\rho & \rho^{2}\\ -{\bar{\rho }}^{2}\rho^{2} & {\bar{\rho }}^{3} & \rho^{3} & -\bar{\rho }\rho \\ -{\bar{\rho }}^{2}\rho^{2} & \rho^{3} & {\bar{\rho }}^{3} & -\bar{\rho }\rho \\ \rho^{4} & -\bar{\rho }\rho^{2} & -\bar{\rho }\rho^{2} & {\bar{\rho }}^{2} \end{array}\right)\left(\begin{array}{c} 4\Phi (H_{1})\\ 8\Phi (H_{2})\\ 8\Phi (H_{3})\\ 16\Phi (H_{4}) \end{array}\right).\;(B2) \end{array}$$
- *For ρ=0.5, let $\alpha =(\kappa_{11}^{cc}(0.5),\kappa_{11}^{ct}(0.5),\kappa_{11}^{tc}(0.5),\kappa_{11}^{tt}(0.5))$, and $\gamma =\frac{1}{2}\kappa_{1}^{2}$. Then,*
  $$\;\alpha =\left\{ \begin{array}{cc} (\gamma ,0,\gamma ,0), & \text{if \textbackslash{};}a\text{ \textbackslash{};is \textbackslash{};an \textbackslash{};ancestor \textbackslash{};of\textbackslash{}; }b,\\ (\gamma ,\gamma ,0,0), & \text{if\textbackslash{}; }b\text{\textbackslash{}; is\textbackslash{}; an\textbackslash{}; ancestor\textbackslash{}; of\textbackslash{}; }a,\\ (\gamma ,0,\gamma ,0), & \text{if\textbackslash{}; }R\text{\textbackslash{}; is\textbackslash{}; collateral\textbackslash{}; and\textbackslash{}; both\textbackslash{}; parents\textbackslash{}; of\textbackslash{}; }a\text{ \textbackslash{};are\textbackslash{}; related\textbackslash{}; to\textbackslash{}; }b,\\ (\gamma ,\gamma ,0,0), & \text{if \textbackslash{};}R\text{ \textbackslash{};is \textbackslash{};collateral\textbackslash{}; and\textbackslash{}; both\textbackslash{}; parents\textbackslash{}; of\textbackslash{}; }b\text{\textbackslash{}; are\textbackslash{}; related\textbackslash{}; to\textbackslash{}; }a,\\ (2\gamma ,0,0,0), & \text{otherwise}. \end{array}\right.$$

Proof (a) Like we did for $H^{*}$ in the proof of Theorem 1, we can express each $\Phi (H_{i})$ as a linear combination of $\kappa_{11}^{cc},\ldots ,\kappa_{11}^{tt}$. In matrix form, the equations work out to be

(B3) $$\left(\begin{array}{c} 4\Phi (H_{1})\\ 8\Phi (H_{2})\\ 8\Phi (H_{3})\\ 16\Phi (H_{4}) \end{array}\right)=\left(\begin{array}{cccc} {\bar{\rho }}^{2} & \bar{\rho }\rho & \bar{\rho }\rho & \rho^{2}\\ \bar{\rho }\rho^{2} & {\bar{\rho }}^{3} & \rho^{3} & {\bar{\rho }}^{2}\rho \\ \bar{\rho }\rho^{2} & \rho^{3} & {\bar{\rho }}^{3} & {\bar{\rho }}^{2}\rho \\ \rho^{4} & {\bar{\rho }}^{2}\rho^{2} & {\bar{\rho }}^{2}\rho^{2} & {\bar{\rho }}^{4} \end{array}\right)\left(\begin{array}{c} \kappa_{11}^{cc}\\ \kappa_{11}^{ct}\\ \kappa_{11}^{tc}\\ \kappa_{11}^{tt} \end{array}\right),$$

The matrix on the right-hand side is invertible for ρ≠0.5, yielding the stated solution. (b) Suppose first that *a* is an ancestor of *b*, and that *a* and *b* share one IBD allele at each locus. Since *b* is noninbred, *a* can only be related to one of *b*’s parents, from which *b* must inherit both alleles. Thus *b*’s alleles are *in cis*, implying that $\kappa_{11}^{ct}=\kappa_{11}^{tt}=0$. In *a*, on the other hand, *cis*/*trans* are equally likely since the loci are independent (ρ=0.5). In other words, $\kappa_{11}^{cc}=\kappa_{11}^{tc}$. The result, when *a* is an ancestor of *b*, then follows since the phased coefficients sum to $\kappa_{11}(0.5)$, which for any relationship equals $\kappa_{1}^{2}$. The same argument applies in the case where R is collateral and both parents of *a* are related to *b*. By symmetry, the cases where *b* is an ancestor of *a*, or R is collateral and both parents of *b* are related to *a*, are treated similarly. The only remaining case is when R is collateral and exactly one parent of *a* is related to exactly one parent of *b*. This clearly enforces that the IBD alleles are *in cis* in both individuals, so that $\kappa_{11}^{cc}$ is the only nonzero contribution. The result follows. ȃ□

## Notes

*Communicating editor: A. Kern*
